# Prevalence of Intestinal Parasite Infection Among Prison Inmates and Their Associated Risk Factors at Hosanna Town, South–Central Ethiopia: A Cross-Sectional Study

**DOI:** 10.1155/2024/7677743

**Published:** 2024-11-14

**Authors:** Abdulhakim Mussema, Weynshet Tafesse, Leyla Temam

**Affiliations:** ^1^Department of Medical Laboratory Science, Wachemo University, Hosanna, Ethiopia; ^2^Department of Biomedical Science, Hawassa University, Hawassa, Ethiopia

**Keywords:** Ethiopia, infection, prevalence, intestinal parasites, prison inmates, risk factors

## Abstract

**Introduction:** In developing countries, prisoners are one of the marginalized groups most susceptible to intestinal parasite infection due to inadequate living conditions, malnutrition, a lack of potable water, overcrowding, and poor hygiene. Thus, this study is aimed at assessing the prevalence and associated factors of intestinal parasitic infections among inmates of Hosanna town prison.

**Materials and Methods:** An institution-based cross-sectional study was conducted in Hosanna Town's prisoners from June 1 to July 30, 2022, on a total of 420 inmates. The study participants were chosen using a simple random sampling technique. Sociodemographic, sanitation, hygienic, and related information were collected by using pretested questionnaires. In addition, about 5 g of stool sample was collected using a leak-proof plastic stool cup and examined microscopically by using direct wet mount preparation and formalin–ether concentration techniques, and data were analyzed by using SPSS Version 25.

**Results:** This study indicated an overall 39.2% (95% CI: 34.6–44.1) prevalence of intestinal parasites (165/420). *Entamoeba histolytica/dispar* was the most prevalent intestinal parasite found, followed by *Giardia lamblia, Ascaris lumbricoides, Hookworms, Taenia* species, and *Schistosoma mansoni*. About 3.1% of participants had dual parasitic infections. In addition, there is a significant association in multivariable logistic regression analysis between intestinal parasitic infection and various hygiene practices such as not using soap when washing hands after using the toilet ((aOR 1.62 (95% CI: 1.06–2.48)), *p* ≤ 0.027), not regularly washing hands before meals ((aOR 2.83 (95% CI: 1.79–4.46)), *p* ≤ 0.001), poor hand hygiene overall ((aOR 3.18 (95% CI: 2.00–4.99)), *p* ≤ 0.001), not trimming fingernails ((aOR 2.09 (95% CI: 1.29–3.37)), *p* ≤ 0.003), and length of time in prison (aOR: 4.27, 95% CI: 22.62–6.96, *p* = 0.001).

**Conclusions:** The findings of the study indicated that the overall prevalence of intestinal parasitic infections was 39.2%, with 38.8% of the infected individuals falling within the 25–34 age range. Additionally, 67.3% of those infected had been imprisoned for over a year. Furthermore, the personal hygiene status of prisoners was found to be substantially correlated with the presence of parasites. Hosanna town prison should ensure sufficient sanitary supplies, promote personal hygiene, and implement health education. Regular medical checkups and routine nail-clipping sessions are crucial for maintaining cleanliness and reducing parasite transmission among inmates.

## 1. Introduction

Intestinal parasites, which include intestinal helminths and protozoan, continue to pose significant public health problems in developing countries that lack adequate drinking water and sanitation [1]. Globally, intestinal parasites are widespread, with an estimated 3.5 billion world populations affected by intestinal parasitic infections (IPIs), and approximately 450 million suffering from illnesses caused by these infections [2]. Annually, an estimated 10.5 million new cases of IPIs are reported. The most prevalent intestinal parasites include *Trichuris trichiura*, *hookworms*, *Ascaris lumbricoides*, *Schistosoma* species, *Giardia lamblia*, and *Entamoeba histolytica* [3, 4]. These infections are often caused by poor hygiene practices, contaminated water sources, and lack of access to adequate healthcare. In addition to causing acute illness, IPIs can also lead to long-term health problems, such as malnutrition, anemia, and impaired cognitive development in children [3–5].

Good hygiene practices, such as avoiding soil ingestion, using latrine, and washing vegetables with clean water before eating, are effective ways to prevent the transmission of parasite infections. To protect against hookworm infection, people should wear shoes or slippers all the time [6, 7]. Campaign programs should be used to inform the public about the importance of good personal and environmental hygiene, such as washing hands after using the toilets and cooking or eating food [8, 9]. Medications for intestinal parasite infections (IPIs) are available through healthcare facilities, school health programs, and community initiatives aimed at vulnerable populations, and they are very effective in treating intestinal parasites [10].

Prisoners are among the most vulnerable and marginalized groups when it comes to IPI. In developing countries, prison inmates endure harsh conditions that include substandard living conditions, limited access to nutritious food, inadequate supply of clean water, overcrowding, and poor sanitation practices [11–13]. Furthermore, inmates have little control over the conditions in which they live, including inadequate access to healthcare, risky behaviors, reduced immunity due to stress, and insufficient nourishment [13, 14].

Studies conducted in different parts of the world have shown a varied prevalence of IPIs among prison inmates [11–13, 15, 16]. Although several studies have been conducted in Ethiopia, the prevalence of IPIs varies across different regions [11, 15, 17–19]. However, there is a lack of information about IPIs and their associated factors among prison inmates in the southern part of Ethiopia, particularly in the current study area. Generating data on the prevalence of intestinal parasites and their related factors could significantly improve health outcomes for the prison inmates. Therefore, this study is aimed at assessing the prevalence of IPIs and associated risk factors among inmates at the Hosanna town prison.

## 2. Materials and Methods

### 2.1. Study Design and Period

An institution-based cross-sectional study was conducted at Hosanna town prison in Hosanna from June 1 to July 30, 2022. The town is situated 232 km from Addis Ababa, Ethiopia, in the central southern region of the country, with geographic coordinates of 7°33⁣′N latitude and 37°51⁣′E longitude, at an elevation of 2177 m above sea level. Hosanna Prison is the only correctional facility within the town. During the data collection period, there were a total of 1446 inmates. Among these 1353 were males. During the study period, between 9 and 43 prison inmates were found across 26 cells.

### 2.2. Study Population, Eligibility Criteria, and Study Variables

The study involved a group of inmates at Hosanna town prison who had not received antiparasitic treatment in the 2 weeks leading up to data collection. Individuals with serious illnesses who were unable to communicate during this time were excluded from the study. The dependent variable of the research was the presence of intestinal parasites, while the independent variables included sociodemographic characteristics, the sanitation and hygiene conditions of the inmates, and other related factors.

### 2.3. Sample Size Determination and Sampling Technique

The sample size was determined using a single population proportion formula (*n* = ((*Zα*/2)^2∗^ *P* (1 − *P*))/*d*^2^) [20] by assuming a 95% confidence level of *Zα*/2 = 1.96, absolute desired precision (*d*) = 5%, and proportion IPIs of 48.1% among inmates of Arba Minch prison [15]. By considering a nonresponse rate of 10%, the final sample size was found to be 423 participants, approximately 30% of the total prisoner population. We used a simple random sampling technique to recruit study subjects.

### 2.4. Data Collection

Information concerning the sociodemographic, sanitation, hygienic, and other characteristics of study participants were collected by using a pretested questionnaires. We developed the questionnaires by thoroughly reviewing existing literature and similar studies [11, 15, 21]. Hand cleanliness was assessed by visually inspecting hands for apparent dirt, grime, or residues by trained data collectors. Hands should be clean and free of soil, contaminants, and residues [22].

### 2.5. Stool Specimen Collection, Processing, and Examination

After receiving the proper instructions from specimen collectors, the prison inmates collected about 5 g of stool sample using leak-proof plastic stool cup and labeled with specific code number. The stool sample was transported immediately to the laboratory of Hosanna town prison clinic and examined macroscopically and microscopically. Macroscopic evaluation of the fecal samples determined their consistency as watery, bloody, or formed prior to microscopic analysis. In the prison clinic laboratory, direct wet mount preparations were made within an hour using normal saline and identified parasitic ova, motile trophozoites, and/or cysts of intestinal parasites, while a stool specimen's sensitivity was enhanced with the addition of 10% formalin for microscopic examinations and formal ether sedimentation using 10 mL of normal saline. The suspension was centrifuged at 2500 rpm for 5 min. The sediment was centrifuged at 2500 rpm for 5 min, washed with a milliliter of 10% formalin, and then, an additional 2 mL ethyl acetate was added. The centrifuge ran at 1500 rpm for 10 min. The top layers were drained off, leaving sediment mixed and present in the bottom. A slide was prepared for microscopic analysis by applying a wet mount of the suspension to identify the different stages of intestinal parasites. We also used an iodine solution for the identification of cysts of intestinal protozoan parasites. We applied 1–2 drops of the sediment suspension and examined it using magnifications ranging from 100x to 400x to observe the sediments under a microscope [23]. Data generated from the laboratory results and questionnaires of similar participants were collected together for analysis.

### 2.6. Data Quality Assurance

Stool specimens and data were collected by trained data collectors. The data collection format of each data collector was checked daily for completeness of missed or other relevant information on meetings and supervision during data collection as well as by the principal investigators. The quality of normal saline and formal ether concentration was checked by the positive preserved sample in the laboratory. Moreover, the investigators used the standard operational procedure (SOP) for the microscopic examination of intestinal parasites. Special emphasis was given for diarrhea stool samples. To avoid misdiagnoses, the macroscopic features of stool were studied to establish the cause of acute diarrhea. In addition to this, a formed and semiformed stool was preserved by formalin for further examination using a microscope. Furthermore, selected slides underwent re-examination and cross-checking by examiners and the supervisor.

### 2.7. Ethical Considerations

This study was conducted after approval by the Research Review Committee of Wachemo University, Department of Medical Laboratory Science with the reference number of MLS432/2014. Then, permission was obtained from the Hosanna town prison administrative. Written informed consent was also obtained from study participants. The aim of the study and their rights of participation were explained through an information sheet. For every confirmed infection identified through laboratory analysis, the study participants received treatment with antiparasitic medications.

### 2.8. Data Analysis

Data was entered into EpiData and cleaned and exported to Excel and SPSS software Version 25 for analysis. Descriptive statistics (frequency tables and cross-tabulations) were used in the analysis. Variables with a *p* value less than 0.25 from the bivariate analysis were chosen and included in the final multiple logistic regression model to identify factors independently associated with the risk of IPI. The multivariable logistic regression model was used to identify the factors associated with parasitic infections. The finding was presented using adjusted odds ratio (aOR) and their 95% confidence interval. Chi-square and odds ratios were computed and a *p* value ≤ 0.05 at a 95% confidence interval was considered statistically significant.

## 3. Results

### 3.1. Sociodemographic Characteristics of the Inmates

A total of 420 prison inmates were enrolled in this study, with a response rate of approximately 99.3%. There were no incomplete data provided by participants in this study. However, it is worth noting that one participant did not provide a stool specimen, and two prisoners were refused permission to participate. Of the total participants, 394 (93.8%) were male, and 26 (6.2%) were female. The mean age of all study participants was 29.98 years (± 10.2 years), with an age range of 19 to 65 years. Among the participants, 234 (55.7%) were married, while 243 (57.9%) came from rural backgrounds. Additionally, 112 participants (26.7%) had not attended school, while 156 participants (37.1%) were unemployed before their imprisonment (Table 1).

### 3.2. Sanitation, Hygienic, and Clinical Characteristics of the Inmates

Almost three-fourths of the inmates (319 or 76%) reported a habit of washing their hands after using the toilet; however, more than half (225 or 53.6%) did not wash their hands with soap. Approximately 128 inmates (30.5%) had untrimmed fingernails. Nearly half (189, or 45%) of the inmates reported sleeping in a group. Additionally, 55.2% of the inmates did not have a waste disposal container in their room. Furthermore, 18% of the study participants had chronic diseases (Table 2).

### 3.3. The Prevalence of Intestinal Parasite Among Prison Inmates

**Figure 1:**
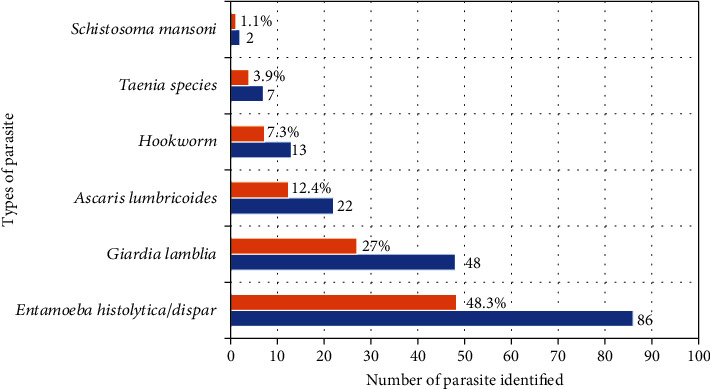
Prevalence of intestinal parasites among inmates of Hosanna town prison, South Ethiopia, 2022.

The study's results indicated that the overall prevalence of IPIs was 39.2% (165/420) (95% CI: 34.6–44.1) (Table 3). Among those infected, 38.8% were in the 25–34 age group, while 26.7% belonged to the 18–24 age group. Additionally, 56.4% of the infected participants came from rural communities. Moreover, 67.3% of the infected participants had been imprisoned for 1 and/or more than 1 year. This statistic indicates that a substantial majority of the participants in the study who tested positive for IPIs had a history of imprisonment lasting 1 year or more. The length of stay in prison was significantly associated with IPI with *X*^2^ of 18.470 (Table 4). This suggests a potential link between incarceration and the likelihood of contracting such infections. Furthermore, 34.6% (9/26) of female participants were found to be infected with intestinal parasites, while 39.59% (156/294) of male participants were similarly infected with IPIs (Table 4). The most prevalent intestinal parasite was *E. histolytica/dispar*, found in 48.3% of cases, followed by *G. lamblia* at 27% and *Ascaris lumbricoides* at 12.4%. Mixed infections were observed in 3.1% (*n* = 13) of the prison inmates (Table 3 and Figure 1).

### 3.4. Factors Associated With IPI of Inmates

In this study, both bivariate and multivariable analyses were conducted on selected variables suitable for logistic regression analysis. Variables with a *p* value below 0.25 from the bivariate analysis were selected and incorporated into the final multiple logistic regression model to determine risk factors independently linked to the risk of IPI. The multivariable analysis revealed a significant association between IPIs and the practice of handwashing without soap after using the toilet (aOR: 1.62, 95% CI: 1.06–2.48, *p* = 0.027), hand washing practices before meals (aOR: 2.83, 95% CI: 1.79–4.46, *p* = 0.001), hand hygiene status (aOR: 3.18, 95% CI: 2.00–4.99, *p* = 0.001), fingernail trimming status (aOR: 2.09, 95% CI: 1.29–3.37, *p* = 0.003), and length of time in prison (aOR: 4.27, 95% CI: 22.62–6.96, *p* = 0.001) (Table 5).

## 4. Discussions

The overall goal of the study is to gain a better understanding of how widespread IPIs are in this specific population and what factors contribute to these infections. This information can be crucial for developing effective prevention and treatment strategies, improving inmate health, and potentially informing public health policies related to correctional facilities. This study found that 39.2% (95% CI: 34.6–44.1) of the Hosanna town prison inmates' had IPIs. In comparison, the results of a comparable study conducted among prisoners, 26.5% in Malaysia [24], 20.2% in Brazil [25], 24.7% in Kenya [16], 15.7% in Spain [13], 14.2% in Nigeria [26], 6% in Nepal prison [27], and 33.5% in a Guinean Prison [12] show that the current prevalence is relatively high in Hosanna town inmates. The high prevalence of IPIs among Hosanna Town inmates might be due to poor hygiene, insufficient access to soap, water, and hand sanitizers, and substandard living conditions including inadequate sanitation facilities and overcrowding.

Comparable prevalence reports have shown that it is 39.35% in Cameroon [28], 38.2% in Ghana [29], and 42.6% in Mekele, Ethiopia, prisons [11]. The comparable reported prevalence rates in Cameroon, Ghana, and Mekele, Ethiopia, studies indicate that IPIs are a common health issue across these regions. This suggests that such infections may be a widespread public health concern in similar socioeconomic and environmental contexts. On the other hand, the higher prevalence of 48.1% in Arba Minch, Ethiopia [15], 68.1% in Shewa Robit, Ethiopia [17], 69.3% in Côte d'Ivoire [24], and 48.8% in Nigeria was reported [30]. The higher prevalence rates observed in certain regions compared to the current study could be due to a combination of methodological differences, environmental conditions, socioeconomic factors, cultural practices, population dynamics, and temporal variations [11, 26, 29].

The higher prevalence of *E. histolytica/dispar* (48.3%) and *G. lamblia* (27%) in the current study and other similar studies [11, 15], compared to helminths, can be attributed to the relatively simple life cycles of *E. histolytica* and *G. lamblia.* These protozoa are resistant to environmental conditions and can survive for extended periods outside their hosts, which facilitates their transmission. They are often spread through contaminated food or water, making them more prevalent in areas with poor sanitation and hygiene practices. Additionally, the study was conducted in June and July, a time when protozoan infections may peak during the rainy season when water sources are more likely to be contaminated [31, 32]. Helminthic infections, although still a concern in this study population, tend to be less common among prison inmates compared to protozoan IPIs. This might be primarily because helminths often require specific environmental conditions for transmission, such as contaminated soil or water or contact with infected animals. Regular cleaning and waste management can limit exposure to soil-transmitted helminths in prison settings.

In this study, the majority of double IPIs involve *E. histolytica/dispar* with *G. lamblia* and *A. lumbricoides* among 3.1% of coinfected inmates, which is comparable with other similar study findings [11, 15–17]. Prison populations, with their overcrowding, poor sanitation, and restricted healthcare, make them susceptible to a range of infectious diseases, including mixed IPIs. The high prevalence of double-infection with *E. histolytica/E. dispar* and *G. lamblia* might indicate their spreading inside these institutional environments. Protozoa are instantly contagious when taken orally, while helminths may take some time to become contagious. For this reason, autoinfection with *E. histolytica*/*E. dispar* may be prevalent.

The majority of sociodemographic and clinical factors did not show a significant association with IPIs among prisoners. However, there were statistically significant associated risk factors with certain hand hygiene practices: washing hands without soap after using the toilet (aOR: 1.62, 95% CI: 1.06–2.48, *p* = 0.027), not regularly washing hands before meals (aOR: 2.83, 95% CI: 1.79–4.46, *p* = 0.001), having poor hand hygiene (aOR: 3.18, 95% CI: 2.00–4.99, *p* = 0.001), having untrimmed fingernails (aOR: 2.09, 95% CI: 1.29–3.37, *p* ≤ 0.003) and length of time in prison (aOR: 4.27, 95% CI: 22.62–6.96, *p* = 0.001). These findings are consistent with research reported elsewhere [2, 11, 18, 29]. These significant associations highlight the critical role that hand hygiene plays in infection prevention, particularly in settings like prisons where the risk of outbreaks can be significant.

This study indicated that the length of time individuals spent in prison is significantly correlated with the prevalence of IPIs (*p* = 0.001). This means that longer imprisonment may increase the risk of infection. This finding may raise important public health concerns. It suggests that the conditions in prisons may contribute to a higher risk of such infections, possibly due to factors like overcrowding, inadequate sanitation, and limited access to healthcare services [2, 6].

Moreover, this study indicates that IPIs are particularly prevalent among young adults, with 38.8% of infected individuals aged 25–34 and 26.7% aged 18–24. This suggests that younger adults may be more vulnerable due to factors such as lifestyle choices or limited health education. Furthermore, a notable 56.4% of the infected individuals hailed from rural communities. This statistic raises important questions about the health infrastructure and resources available in these areas. Rural communities often face challenges such as limited access to clean water, inadequate sanitation facilities, and fewer healthcare resources, all of which can contribute to higher rates of parasitic infections. Gender differences were observed, with 34.6% of female participants and 39.59% of male participants testing positive for IPIs. While the disparity is not large, it points to a slightly higher infection rate among males, which could be influenced by behavioral or environmental factors.

Intestinal parasites can be transmitted to consumers through food contaminated by unclean hands or flies that have landed on both the food and feces [2, 6, 9, 18]. The untrimmed fingernails could be a contributing factor to parasitic infection or ingestion due to unsanitary conditions [6, 29]. This observed prevalence significantly concerns all stakeholders. Addressing these hygiene practices through education and improved access to hygiene resources could potentially reduce infection rates in such populations [33, 34]. Organize regular, supervised nail clipping sessions where prison officers or medical staff provide nail clippers to inmates and oversee their use [35]. This ensures the maintenance of hygiene standards and reduces the cross-contamination of parasites among prisoners through untrimmed fingernails.

## 5. Limitation of the Study

Due to a lack of resources, we were unable to differentiate between *E. dispar* and *E. histolytica* using molecular techniques. Furthermore, we did not employ any additional approaches that are specific to certain intestinal parasites, such as the modified Ziehl–Neelsen staining methods or the Kato–Katz technique.

## 6. Conclusions and Recommendations

As a result, intestinal parasites are quite prevalent among prisoners in Hosanna Town. The presence of parasites in the stool of inmates was significantly associated with their personal hygiene and nail-trimming practices. Therefore, it is essential for Hosanna town prison to provide appropriate sanitary supplies, including soap, and to promote good personal hygiene practices. Additionally, implementing regular medical check-up programs and comprehensive health education focused on personal hygiene may be crucial for preventing and controlling parasite transmission. Furthermore, establishing regular nail-clipping sessions can help maintain cleanliness standards and reduce the spread of parasites among prisoners due to untrimmed fingernails.

## Figures and Tables

**Table 1:** Sociodemographic characteristics of prison inmates at Hosanna town prison, Southern Ethiopia, 2022 (*n* = 420).

**Characteristics**		**n** ** (%)**
Age	18–24	98 (23.3)
	25–34	162 (38.6)
	35–44	94 (22.4)
	≥ 45	66 (15.7)

Gender	Female	26 (6.2)
	Male	394 (93.8)

Marital status	Married	234 (55.7)
	Single/widowed	186 (44.3)

Residence	Urban	177 (42.1)
	Rural	243 (57.9)

Educational status	No formal education/did not attend school	112 (26.7)
	Primary school	214 (51.0)
	High school	70 (16.7)
	Above high school	24 (5.7)

Occupation	Farmer	206 (49.0)
	Civil servant	27 (6.4)
	Merchant	14 (3.3)
	Housewife	17 (4.0)
	Unemployment	156 (37.1)

Monthly income (birr)	<3000	154 (36.7)
	≥3000	89 (21.2)
	No income	177 (42.1)

Length of time in prison	Less than 1 year	192 (45.7)
	One and/or more than 1 year	228 (54.3)

**Table 2:** Sanitation, hygienic, and clinical characteristics of prison inmates of Hosanna town prison, Southern Ethiopia, 2022 (*n* = 420).

**Characteristics**		**n** ** (%)**
Number of prisoners in the room	< 10	203 (48.3)
	≥ 10	217 (51.7)

Hand washing habit after toilet	No	101 (24.0)
	Yes	319 (76.0)

Hand washing habit after handling soil	No	229 (54.5)
	Yes	191 (45.5)

How often do you wash your hands after the toilet?	Sometimes	234 (73.4)
	Always	85 (26.6)

How do you wash your hands after the toilet?	Without soap	225 (79.9)
	Water and soap	64 (20.1)

Hand washing before meal	Sometimes	228 (54.3)
	Always	192 (45.7)

How you eat fruit	Without washing	169 (40.2)
	After washing	251 (59.8)

Meat eating habit	Raw	209 (49.8)
	Cooked	211 (50.2)

Status of hand hygiene	Clean	228 (54.3)
	Not clean	192 (45.7)

Fingernail trimming status	Untrimmed	128 (30.5)
	Trimmed	292 (69.5)

How often do you wear shoes?	Sometimes	191 (45.5)
	Always	229 (54.5)

Source of drinking water	Pipe water	190 (45.2)
	Tanker	230 (54.8)

Is there a waste disposal container in your room?	No	232 (55.2)
	Yes	188 (44.8)

Style of sleeping	By group	189 (45.0)
	Individually	231 (55.0)

Type of bathroom	By group	289 (68.8)
	Individually	131 (31.2)

History of bloody diarrhea	No	230 (54.8)
	Yes	190 (45.2)

Chronic disease	No	344 (81.9)
	Yes	76 (18.1)

Type of chronic disease	Diabetes	18 (4.3)
	HIV/AIDS	10 (2.4)
	Heart disease	13 (3.1)
	Gastritis	38 (9.0)
	Hypertension	8 (1.9)

*Note:* Chronic disease: diabetes mellitus, hypertension, and heart disease.

**Table 3:** Prevalence of intestinal parasites among inmates of Hosanna town prison, South Ethiopia, 2022.

**Identified intestinal parasites**	**Frequency**	**Percentage (%)**
*Entamoeba histolytica/dispar*	75	17.9
*Giardia lamblia*	41	9.8
*Ascaris lumbricoides*	17	4.0
*Hookworms*	11	2.6
*Taenia* species	6	1.4
*Schistosoma mansoni*	2	0.5
*Entamoeba histolytica/dispar + Giardia lamblia*	6	1.4
*Entamoeba histolytica/dispar + Ascaris lumbricoides*	4	1.0
*Entamoeba histolytica/diyspar + Hookworms*	1	0.2
*Ascaris lumbricoides + Hookworms*	1	0.2
*Giardia lamblia + Taenia* species	1	0.2
Total positive inmates with intestinal parasite	165	39.2

**Table 4:** Distribution of intestinal parasitic infections by age, gender, residence, and length of time among inmates of Hosanna town prison, South Ethiopia, 2022.

**Characteristics**		**Intestinal parasitic infection status**		
		**Yes (%)**	**No (%)**	**X** ^2^ ** (** **p** ** value)**
Age	18–24	44 (26.7)	54 (21.2)	2.225 (0.527)
	25–34	64 (38.4)	98 (38.4)	
	35–44	34 (20.6)	60 (23.5)	
	≥ 45	23 (13.9)	43 (16.9)	

Gender	Female	9 (6.7)	17 (5.5)	0.253 (0.615)
	Male	156 (93.3)	238 (94.5)	

Residence of participant	Urban	72 (41.2)	105 (43.6)	0.249 (0.618)
	Rural	93 (58.8)	150 (56.4)	

Length of time in prison	Less than 1 year	54 (32.7)	138 (54.1)	18.470 (0.001)
	One and/or more than 1 year	111 (67.3)	117 (45.9)	

*Note: X* ^2^: Pearson chi-square.

**Table 5:** Bivariate and multivariable analysis of factors associated with intestinal parasitic infection of the inmates at Hosanna town prison, South Ethiopia, 2022.

**Characteristics**		**Intestinal parasitic infection status**		**Bivariate analysis**	**Multivariable analysis**	
		**Positive (%)**	**Negative (%)**	**cOR (95% CI)**	**aOR (95% CI)**	**p** ** value**
Age	18–24	44 (26.7)	54 (21.2)	1.52 (0.80–2.90)	1.13 (0.55–2.33)	0.737
	25–34	64 (38.4)	98 (38.4)	1.22 (0.67–2.22)	1.42 (0.74–2.74)	0.291
	35–44	34 (20.6)	60 (23.5)	1.06 (0.55–2.05)	1.47 (0.71–3.04)	0.297
	≥ 45	23 (13.9)	43 (16.9)	1	1	

Length of time in prison	Less than 1 year	54 (32.7)	138 (54.1)	1	1	
	One and/or more than 1 year	111 (67.3)	117 (45.9)	4.23 (2.61–3.65)	4.27 (2.62–6.96)	0.001⁣^∗^

Number of prisoners in the room	< 10	91 (55.2)	112 (43.9)	1	1	
	≥ 10	74 (44.8)	143 (56.1)	1.50 (1.06–2.33)	1.46 (0.85–2.52)	0.171

Hand washing after toilet	Without soap	102 (48.2)	123 (61.8)	1.74 (1.1–2.59)	1.62 (1.06–2.48)	0.027⁣^∗^
	Water and soap	63 (51.8)	132 (38.2)	1	1	

Hand washing before meal	Sometimes	107 (64.8)	121 (47.5)	2.04 (1.37–3.06)	2.83 (1.79–4.46)	0.001⁣^∗^
	Always	58 (35.2)	134 (52.5)	1	1	

How you eat fruit	Without washing	75 (45.5)	94 (36.9)	1.43 (0.96–2.13)	1.02 (0.62–1.66)	0.945
	After washing	90 (54.5)	161 (63.1)	1	1	

Status of hand hygiene	Not clean	111 (67.3)	117 (45.9)	2.43 (1.61–3.65)	3.18 (2.00–4.99)	0.001⁣^∗^
	Clean	54 (32.7)	138 (54.1)	1	1	

Fingernail trimming status	Untrimmed	128 (77.6)	164 (64.3)	1.92 (1.23–3.00)	2.09 (1.29–3.37)	0.003⁣^∗^
	Trimmed	37 (22.4)	91 (35.7)	1	1	

Abbreviations: aOR, adjusted odds ratio; CI, confidence interval; cOR, crude odds ratio.

⁣^∗^Statically significant.

## Data Availability

The data that support the findings of this study are available on request from the corresponding author. The data are not publicly available due to privacy or ethical restrictions.

## Nomenclature

AOR: adjusted odds ratio
CI: confidence interval
IPI: intestinal parasitic infections
